# Overexpression of *PtrABF *gene, a bZIP transcription factor isolated from *Poncirus trifoliata*, enhances dehydration and drought tolerance in tobacco via scavenging ROS and modulating expression of stress-responsive genes

**DOI:** 10.1186/1471-2229-10-230

**Published:** 2010-10-25

**Authors:** Xiao-San Huang, Ji-Hong Liu, Xue-Jun Chen

**Affiliations:** 1 National Key Laboratory of Crop Genetic Improvement, Key Laboratory of Horticultural Plant Biology of Ministry of Education, Huazhong Agricultural University, Wuhan 430070 China; 2 Yunnan Academy of Tobacco Agricultural Sciences, Yuxi 653100, China

## Abstract

**Background:**

Drought is one of the major abiotic stresses affecting plant growth, development and crop productivity. ABA responsive element binding factor (ABF) plays an important role in stress responses via regulating the expression of stress-responsive genes.

**Results:**

In this study, a gene coding for ABF (*PtrABF*) was isolated from *Poncirus trifoliata *(L.) Raf. *PtrABF *had a complete open reading frame of 1347 bp, encoding a 448 amino acid peptide, and shared high sequence identities with ABFs from other plants. PtrABF was subcellularly targeted to the nucleus, exhibited transactivation activity in yeast cell and could bind to ABRE, supporting its role as a transcription factor. Expression levels of *PtrABF *were induced by treatments with dehydration, low temperature and ABA. Ectopic expression of *PtrABF *under the control of a CaMV 35S promoter in transgenic tobacco plants enhanced tolerance to both dehydration and drought. Under dehydration and drought conditions, the transgenic plants accumulated lower levels of reactive oxygen species compared with wild type, accompanied by higher activities and expression levels of three antioxidant enzymes. In addition, steady-state mRNA levels of nine stress-responsive genes coding for either functional or regulatory proteins were induced to higher levels in the transgenic lines with or without drought stress.

**Conclusions:**

*PtrABF *is a bZIP transcription factor and functions in positive modulation of drought stress tolerance. It may be an important candidate gene for molecular breeding of drought- tolerant plants.

## Background

Plants are frequently threatened by drought that affects growth and development, productivity and quality [1]. Therefore, improvement of drought tolerance has been the subject of intense studies via either traditional breeding or biotechnology-mediated approach. As a first step towards improvement of drought tolerance via genetic engineering in plant, genes with functions in drought tolerance should be cloned and characterized. It has been established that plants respond and adapt to drought stress through modifying transcriptional levels of a large number of genes [2,3]. These genes can be generally classified into two main groups based on their products, effector molecules or regulator molecules, which are also correspondingly designated as delayed-response genes or early-response genes based on their response timing [4-6]. Products of the former group function directly in protecting cells against damage derived from stresses and sustaining cell viability, and the latter is composed of regulatory proteins, such as transcription factors (TFs), protein kinases and protein phosphatases, that are responsible for transducing stress signaling and regulating expression of stress-responsive genes [5].

TF constitutes the key regulon that plays crucial roles in various biological processes, including stress response, through binding to the *cis*-acting element in the promoters of target genes [7]. ABA-responsive element (ABRE, PyACGTGGC), an important *cis*-element, is implicated in the transcriptional regulation of ABA- and/or stress-responsive genes [8]. In plants, ABRE binding factors (ABFs)/ABRE binding proteins (AREBs) are basic region/leucine zipper (bZIP) class TFs [9,10] that interact with ABRE. ABF/AREB family TFs have been cloned and characterized in *Arabidopsis thaliana *and other plants [11-15]. In the *Arabidopsis *genome, nine ABF/AREB homologues have been identified so far [16,17]. Up-regulation of ABF/AREB family members by various abiotic stresses, such as drought, high salinity, cold, anoxia, and by ABA in different plants suggests that they are involved in ABA and/or stress signaling [11,12,14,16,17].

TFs are known as master switch in plant stress response, and genetic transformation of the genes encoding TFs has been suggested as a possible approach for engineering stress tolerance as manipulation of a single TF can alter expression of a wide array of target genes [7]. Genetic transformation of ABF/AREB has been shown to render stress tolerance. For example, constitutive overexpression of *AREB1/ABF2*, *ABF3 *or *ABF4 *in *Arabidopsis *resulted in enhanced drought tolerance [14,18]. In another work, interestingly, overexpression of *ABF2 *in *Arabidopsis *was found to promote tolerance to drought, high salt, heat and oxidative stresses [19]. Later, ABF/AREB family members have been transformed into lettuce and *Agrostis mongolica*, and the transgenic plants were more tolerant to drought and cold/heat stresses than wild type [20,21]. All of these successful examples indicate that genetic manipulation of ABF/AREB family members can be of significance for modifying plant stress response [22].

Although ABF/AREB members have been comprehensively studied in the model plants, information concerning their counterparts in woody plants is relatively lacking. Trifoliate orange (*Poncirus trifoliata *(L.) Raf), an important rootstock for citrus, is drought intolerant, which has restricted its use in regions with water deficit. A raised question is whether or not the ABF homologue in trifoliate orange can function in stress tolerance. On the other hand, it was noticed that limited information is available concerning physiological and/or molecular basis on enhanced stress tolerance in the transgenic plants overexpressing an ABF gene in previous reports. Therefore, we tired to clone an ABF gene from trifoliate orange, and functionally characterize its role in dehydration/drought tolerance. In addition, accumulation of reactive oxygen species (ROS) and expression of stress-responsive genes in the transgenic plants were illustrated.

## Results

### Cloning and bioinformatics analysis of *PtrABF*

**Figure 1:**
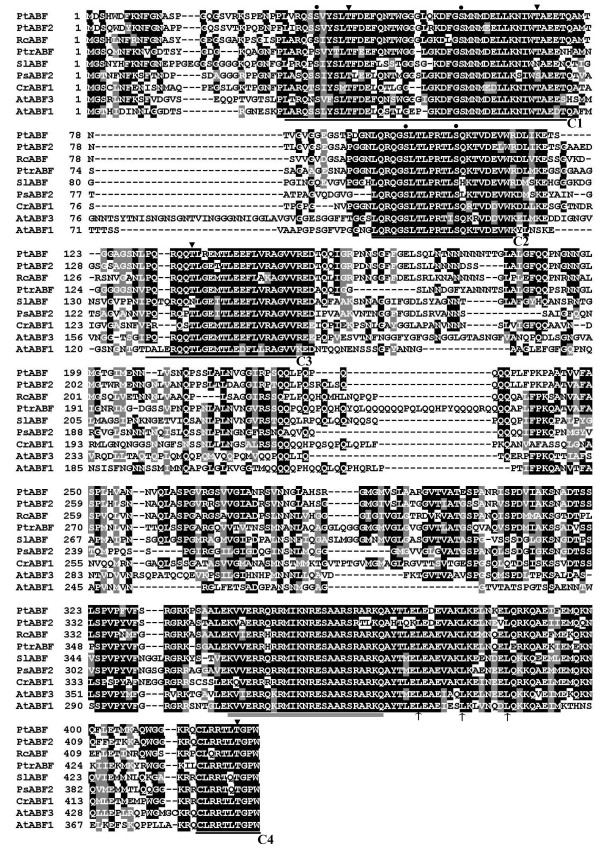
**Multiple alignments of the deduced amino acid sequence of PtrABF and those of *Arabidopsis thaliana *(AtABF1, NP_564551.1; AtABF3, NP_567949.1), *Catharanthus roseus *(CrABF1, AF329450_1), *Populus suaveolens *(PsABF2, ABF29696.1), *Populus trichocarpa *(PtABF, XP_002313119.1), *Ricinus communis *(RcABF, XP_002518757.1), *Solanum lycopersicum *(SlABF, AAS20434.1) *and Populus trichocarpa *(PtABF2, ABN58425.1)**. Black and gray shaded backgrounds indicated that the amino acids were identical or similar to PtrABF, respectively. The basic region and the three heptad leucine repeats, two important bZIP signatures, are shown by double line and arrows, respectively. C1, C2, C3 and C4 are conserved regions containing residues of serine ('•') and threonine ('▼').

**Figure 2:**
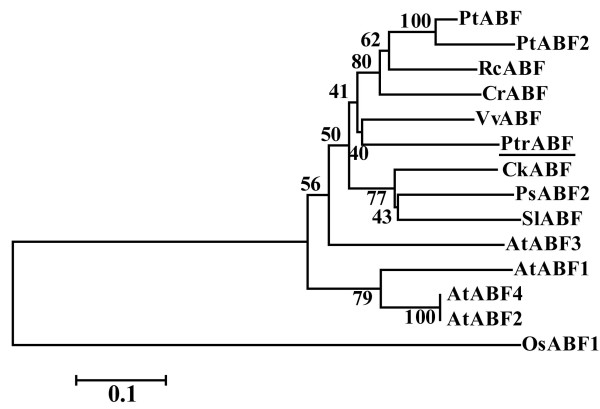
**A phylogenetic tree constructed based on the amino acid sequences of PtrABF (underlined) and other 12 ABFs, including AtABF1, AtABF3, PtABF, PsABF2, SlABF, RcABF, PtABF2, CrABF (AF329450_1), VvABF (CAN64991.1), CkABF (ABG90380), AtABF2 (NP_849777), AtABF4 (NP_566629)**. The numbers beside the branches represent bootstrap values based on 1000 replications, and the relative amount of change along branches is indicated by the scale bar.

Eighteen sequences were found in the search of the citrus EST database, which were successfully combined into a contig. RT-PCR using the gene specific primers (GSP1) designed based on the contig gave rise to a single band. Sequencing and bioinformatics analysis showed that the cDNA, 1511 bp in length, contained a 1347-bp open reading frame (ORF), along with a 35-bp 5' untranslated region (UTR) and a 129-bp 3' UTR. The cDNA, designated as *PtrABF *(*Poncirus trifoliata ABF*), encodes a predicted polypeptide of 448 amino acids with a calculated molecular weight of 48 kDa and a pI of 9.66. This gene has been deposited in NCBI under the accession number of HM171703. Multiple alignments between PtrABF and eight other ABF proteins revealed high sequence identity among each other (Figure 1). At the C-terminus of PtrABF there was a bZIP signature (from position 366 to 424, E-value 3.98e-09) composed of a 25-amino acid basic region and a leucine zipper containing three leucine repeats (at positions 396, 403 and 410) that were spaced from each other by seven amino acids. The proteins had four highly conserved regions, three at the N-terminus (designated as C1, C2 and C3) and one at the end of C-terminus (C4). The phylogenetic tree constructed based on amino acid sequences of PtrABF and ABFs from other 13 representative plants showed that PtrABF was most closely related to its counterpart of grape, while it is most distant to rice (Figure 2).

### Expression pattern of *PtrABF *under various treatments

**Figure 3:**
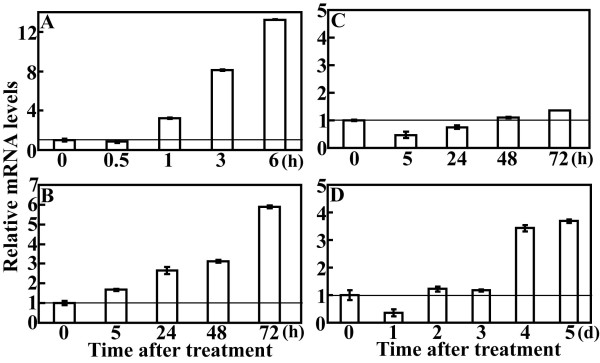
**Time-course expression levels of *PtrABF *in trifoliate orange shoots under different treatments with dehydration (A), low temperature (B), salt (C) and ABA (D)**. Total RNA extracted from leaves of the treated shoots sampled at the indicated time was reverse-transcribed to synthesize cDNAs, which were used for quantitative real time PCR analysis. For each treatment, the expression level at time point 0 was defined as 1.0, and data represented means ± SE of three replicates.

In plants, an important feature of *ABFs *is the induction of expression by various abiotic stresses. In order to know if *PtrABF *acts in the similar way, expression profiles of *PtrABF *under different treatment (stresses or ABA) were assessed via QRT-PCR. The transcript level of *PtrABF *began to accumulate 1 h after dehydration, and increased progressively until the end of treatment, which was more than 13 folds of the initial level (Figure 3A). Steady-state mRNA of *PtrABF *began to accumulate 5 h after low temperature treatment, and continued to increase until it reached the highest level at 3 d (Figure 3B). When subjected to salt treatment, the transcript of *PtrABF *did not change notably except a slight decrease at 5 h and 1 d (Figure 3C), indicating that PtrABF was not salt-inducible. In the case of ABA treatment, mRNA abundance was first reduced at 1 d, resumed to the basal level during the next two days, and increased at 4 d and maintained thereafter (Figure 3D).

### PtrABF was localized in the nucleus

**Figure 4:**
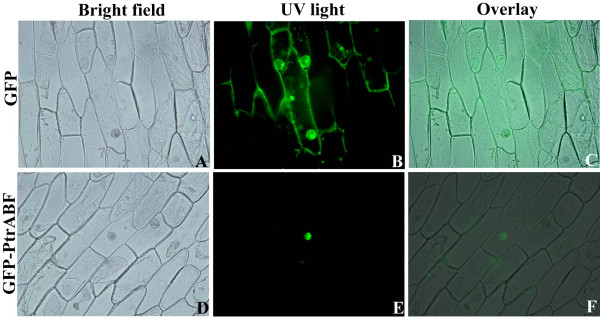
**Subcellular localization of PtrABF via *Agrobacterium*-mediated transformation of onion epidermis**. Fusion construct (PtrABF: GFP) and control plasmid (GFP) were separately transformed into onion epidermal cells via *Agrobacterium *infection. Bright-field images (A, D), fluorescence images (B, E), and the merged images (C, F) of representative cells expressing GFP (A, B and C) or PtrABF: GFP fusion protein (D, E and F) are shown.

Sequence analysis showed that there was a nuclear localization signal (from position 359 to 376) in the basic region of the bZIP DNA binding domain, implying that PtrABF may be localized in the nucleus. In order to verify this, subcellular localization of *PtrABF *was examined by monitoring the GFP fluorescence in the onion epidermis cells transformed with either the fusion construct (PtrABF-GFP) or the control (GFP). When onion cells were transformed with GFP plasmid, green fluorescence signals were observed in the entire cell region (Figure 4A-C). In contrast, fluorescence was exclusively detected in the nucleus of cells transformed with the fusion plasmid (Figure 4D-F), implying that PtrABF is a nuclear protein.

### PtrABF had transactivation activity and could bind to ABRE

**Figure 5:**
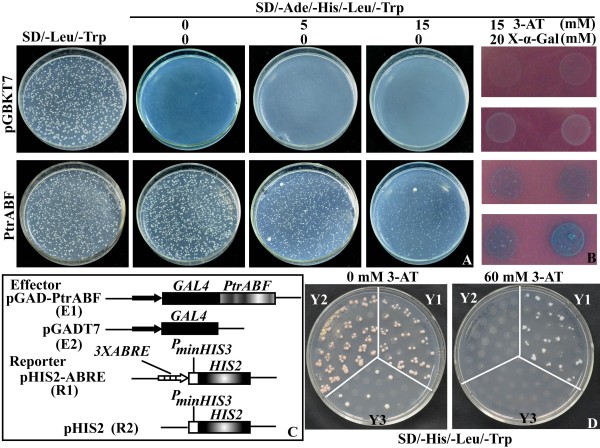
**Analysis of transactivation activity and ABRE binding of PtrABF**. A. Growth of yeast cells transformed with either control vector (pGBKT7) or the fusion vector (pGBKT7: PtrABF) on SD/-Leu/-Trp, SD/-Ade/-His/-Leu/-Trp supplemented with 3-AT (0, 5 and 15 mM). B. Growth of the yeast cells on SD/-Ade/-His/-Leu/-Trp added with 15 mM 3-AT and 20 mM X-α-gal. C. Schematic diagrams of effector plasmids (E1 and E2) and reporter plasmids (R1 and R2) used for yeast one hybrid. D. Growth of yeast cells on SD/-His/-Leu/-Trp supplemented with (60 mM) or without 3-AT. Y1, Y2 and Y3 represent yeast cells transformed with E1 and R1, E2 and R1, E1 and R2, respectively.

Transactivation activity is another defining feature for a transcription factor in addition to nuclear localization. To identify if PtrABF functions as a transcriptional activator the yeast two-hybrid analysis was used. For this purpose, the full-length *PtrABF *coding region was fused to the DNA-binding domain of GAL4 to generate a fusion plasmid, which was transformed into yeast and growth of the cells was compared with those transformed with the control plasmid (pGBKT7) on the same medium. Yeast cells carrying either the control plasmid or the fusion plasmid grew well on SD/-Leu/-Trp medium, indicating that the analysis system was reliable. On the SD/-Ade/-His/-Leu/-Trp medium, the cells transformed with the control plasmid could not grow, whereas those transformed with the fusion plasmid grew normally. The yeast cells transformed with the fusion plasmid could also live on the medium added with 3-AT, but the growth was repressed in a concentration-dependent manner (Figure 5A). In addition, when the yeast cells were cultured on the SD/-Ade/-His/-Leu/-Trp medium added with both 15 mM 3-AT and 20 mM X-α-Gal, only those transformed with the fusion plasmid turned blue (Figure 5B). All of these results indicated that PtrABF had transactivation activity in yeast.

Previous work showed that ABF can bind to the *cis*-element ABRE [8]. In order to investigate whether PtrABF has the same DNA-binding activity, the full length ORF of *PtrABF *was fused to the GAL4 activation domain of vector pGADT7 and the fused construct (pGADT7-PtrABF) was co-transformed with pHIS-ABRE construct containing triple tandem repeats of ABRE into yeast strain Y187 (Figure 5C). Although all of the yeast cells with different constructs could grow on SD/Leu-/Trp-/His medium without 3-AT, only the cells co-transformed with pGADT7-PtrABF and pHIS-ABRE grew normally in the presence of 3-AT, while the growth of transformants containing constructs lacking either PtrABF or ABRE was completely inhibited (Figure 5D), suggesting that PtrABF could bind to the ABRE and activate the reporter gene in yeast.

### Overexpression of *PtrABF *enhances tolerance to dehydration and drought

**Figure 6:**
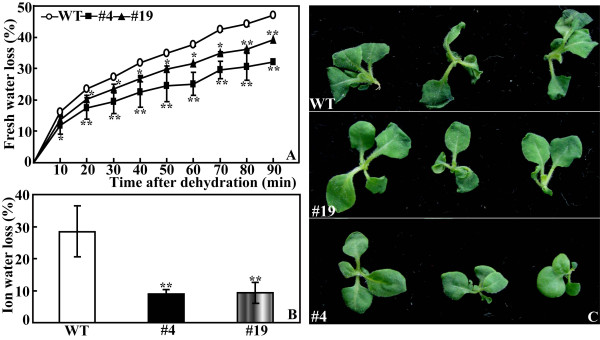
**Phenotype and dehydration tolerance of wild type (WT) and transgenic lines (#4 and #19)**. (A) Time-course fresh water loss of WT, #4 and #19 during a 90-min dehydration. Aerial parts of 35-d-old *in vitro *seedlings were dehydrated at ambient environment, and the fresh weight (FW) was measured at the indicated time. Water loss was calculated by the decrease of FW relative to that at time 0. (B) Electrolyte leakage of WT, #4 and #19 after dehydration for 90 min. * and ** indicate that values of the two transgenic lines were significantly different from those of WT at *P *< 0.05 and *p *< 0.01, respectively. (C) Representative photographs of dehydrated WT (upper panel), #4 (middle panel) and #19 (lower panel).

To investigate the function of *PtrABF*, *Agrobacterium*-mediated transformation of tobacco leaf discs was carried out using a binary vector containing *PtrABF *under the control of 35S promoter of cauliflower mosaic virus (CaMV 35S). Totally, 9 T0 lines were characterized by PCR with primers specific to CaMV 35S and *PtrABF *and *NPTII*, and 7 out of them were confirmed as putative transgenic lines, and overexpression of *PtrABF *in two lines (#4 and #19) was verified by semi-quantitative RT-PCR analysis (data not shown). These two lines and wild type (WT) were subjected to dehydration and drought treatment to investigate the role of *PtrABF *in water stress response. No morphological difference was noted between the transgenic lines and WT (data not shown). When the aerial parts of 5-week-old *in vitro *seedlings were dehydrated, fresh water loss was increased in the two transgenic lines and WT with the progressing of dehydration. However, significantly higher water loss was observed in WT than the two transgenic lines from 10 min onwards (Figure 6A). Although #19 lost less water than #4, the difference was not statistically significant except at 60 and 90 min after dehydration. Electrolyte leakage of #4 was equal to that of #19, both of which were significantly smaller than WT (Figure 6B). When dehydration was completed, leaves of WT exhibited more serious wilting relative to the transgenic lines (Figure 6C).

**Figure 7:**
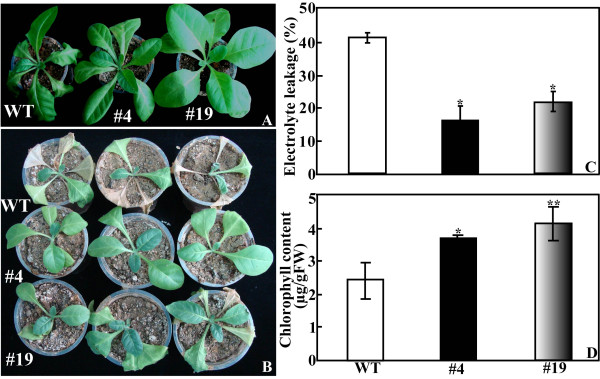
**Phenotype and drought tolerance of wild type (WT) and transgenic lines (#4 and #19)**. (A-B) Representative photographs of potted plants of WT, #4 and #19 that have been exposed to water stress for 7 d (A) and 21 d (B). (C-D) Electrolyte leakage (C) and total chlorophyll content (D) of WT, #4 and #19 after 7 d of water stress. * and ** indicate that values of the two transgenic lines were significantly different from those of WT at *P *< 0.05 and *P *< 0.01, respectively.

Apart from dehydration, long-term water stress (drought) tolerance of the potted plants was also examined by water withholding. Morphological difference became apparent after watering was stopped for 7 d, when WT lost its turgor while #4 and #19 grew better (Figure 7A). When the drought was extended to as long as 3 weeks, phonotypical difference was more dramatic between WT and the transgenic plants, as manifested by presence of more dead leaves in the former (Figure 7B). Electrolyte leakage of #4 (16.7%) and #19 (22.3%) was significantly lower in comparison with 42.3% of wile type (Figure 7C), whereas total chlorophyll of the transgenic lines (3.65 μg g^-1 ^FW for #4 and 4.13 μg g^-1 ^FW for #19) was significantly higher than WT (2.42 μg g^-1 ^FW, Figure 7D). The above results showed that the two transgenic lines were more tolerant to short-term dehydration shock or long-term water stress (drought).

### Accumulation of O_2_- and H_2_O_2 _in the transgenic lines and WT under drought

**Figure 8:**
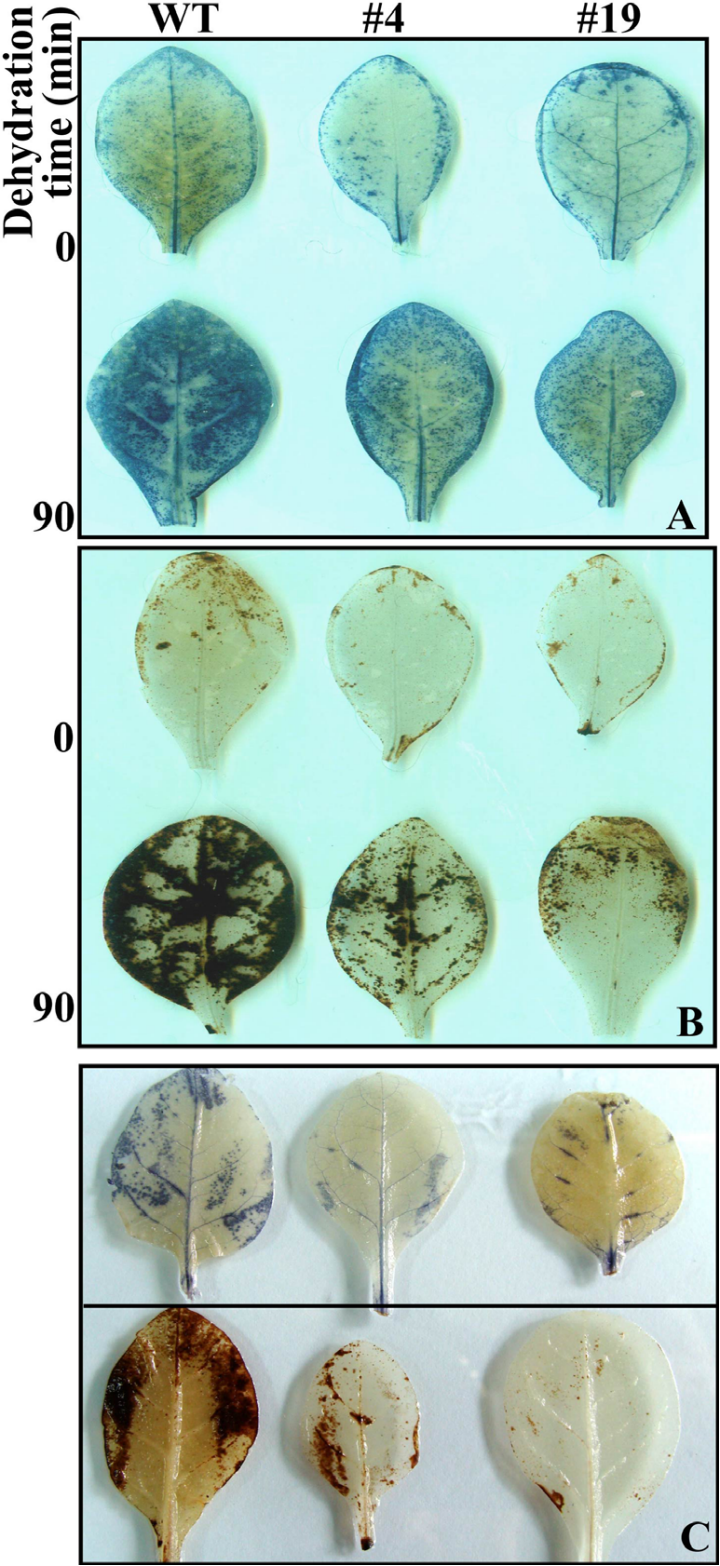
**Histochemical staining by nitro blue tetrazolium (NBT) and diaminobenzidine (DAB) to reveal accumulation of O_2_- and H_2_O_2 _in leaves of wild type (WT), transgenic lines (#4 and #19) subjected to 90-min dehydration or 21-d water stress**. (A-B) Representative photos showing accumulation of O_2_- (A) and H_2_O_2_(B) in leaves before (0) and after 90-min (90) dehydration. (C) Representative photos showing accumulation of O_2_- (upper panel) and H_2_O_2_(lower panel) in leaves of plants after 21- d water stress.

As the transgenic lines were more tolerant to dehydration and drought than wild type, attempts were made to compare accumulation of H_2_O_2 _and O_2_-, two main reactive oxygen species (ROS), in their leaves sampled at the end of water stress. For this purpose, leaves of the transgenic lines and wild type that have been either dehydrated for 90 min or exposed to drought for 21 d were stained with DAB or NBT to reveal *in situ *accumulation of H_2_O_2 _and O_2_-, respectively. Histochemically staining showed that before dehydration, H_2_O_2 _and O_2_- could also be stained, and the transgenic lines had slightly lower ROS levels relative to WT. Dehydration resulted in notable increased of ROS levels in both wild type and the two transgenic lines, whereas WT accumulated remarkably more O_2_- and H_2_O_2 _than both #4 and #19, as less blue (Figure 8A) or brown (Figure 8B) products were observed in the latter two. Similarly, under drought the two transgenic lines accumulated dramatically less O_2_- and H_2_O_2 _than WT (Figure 8C), implying that less ROS was produced in the transgenic lines under the stressful conditions.

### Analysis of activity of antioxidant enzymes and expression levels of the encoding genes in transgenic lines and WT before and after drought

**Figure 9:**
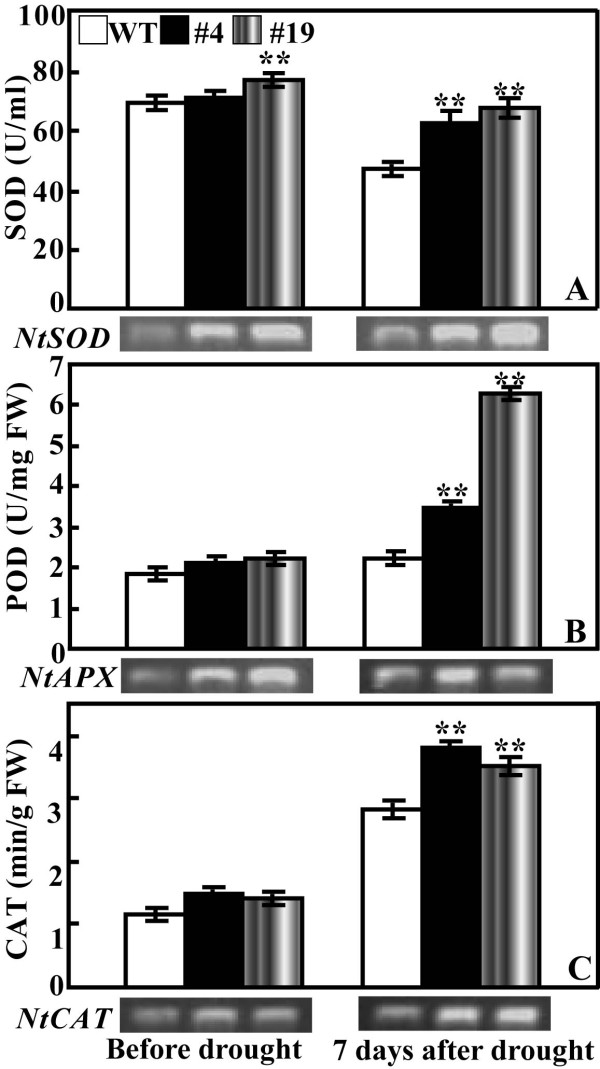
**Activity and expression of antioxidant enzymes (genes) in wild type (WT), transgenic lines (#4 and #19) before and after drought treatment**. (A-C) Activity of SOD (A), POD (B) and CAT (C) of WT, #4 and #19 plants before and after 7-d of drought treatment. Enzymes were extracted and assayed as described in 'Materials and methods'. ** indicates that the values of the two transgenic lines are significantly different those of WT (*P *< 0.01). Expression of the genes encoding the enzymes was examined by RT-PCR and was shown below each graph.

The above-mentioned results demonstrated that the two transgenic lines produced less ROS compared with WT when they were simultaneously stressed. As ROS-scavenging enzymes play critical role in detoxifying ROS activities of three key enzymes, superoxide (SOD), catalase (CAT) and peroxidase (POD), were measured in the two transgenic lines and WT before and 7 d after drought. Before water stress, activities of the three enzymes were higher than those of the control, but the difference was not prominent. Water stress caused decrease of SOD activity, which was significantly lower in WT than in #4 and #19 (Figure 9A). Exposure to drought resulted in slight rise of POD activity in WT, which was notably enhanced in the two transgenic lines. As a result, POD activity of #4 and #19 was 1.5 and 3 folds of that in WT, respectively (Figure 9B). Activity of CAT was augmented in all of the tested samples, while the transgenic lines had significantly higher activities than WT (Figure 9C). All of these showed that activities of the three detoxifying enzyme were higher in the transgenic lines than WT, in reverse proportion to the ROS accumulation of these lines. Examination of the genes encoding SOD, CAT and *APX *showed that before water stress the expression levels of the three genes were higher in the transgenic lines than in WT. Exposure to drought led to induction of these genes in both WT and the transgenic plants, whereas the transcript levels were still higher in the latter except *NtAPX *in #19.

### Expression analysis of stress-responsive genes before and after drought

**Figure 10:**
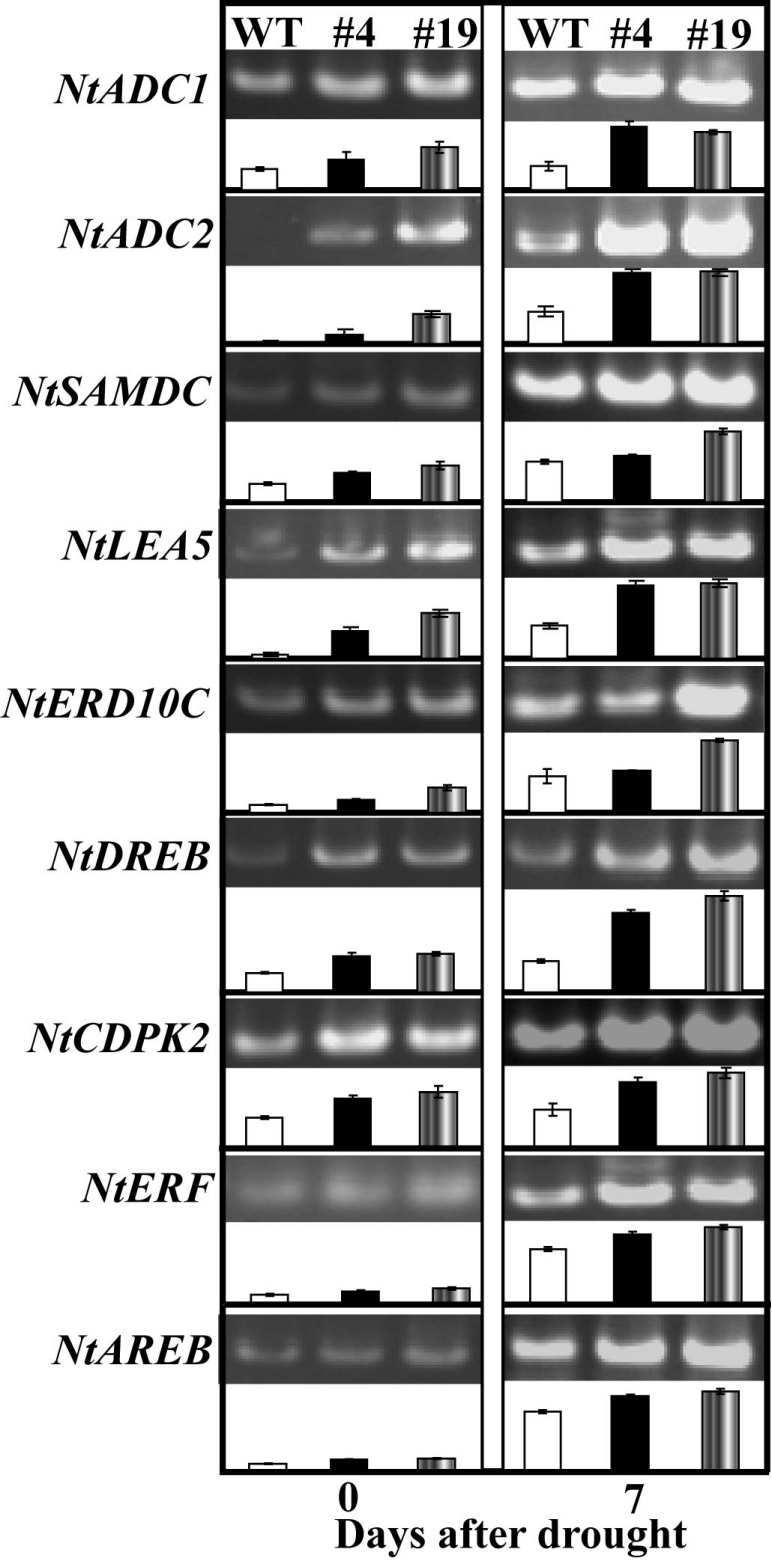
**Expression profiles of the nine stress-responsive genes in wild type (WT), transgenic lines (#4 and #19) before and after drought treatment**. RNA was extracted from leaves sampled at the onset and after 7 d of water stress, and reverse-transcribed to synthesize cDNA, which was used for RT- PCR analysis with primers specific for these genes. mRNA levels of these genes were normalized to the transcripts of *Tubulin *in the same samples. The graph below each gel panel was made based on the means of three independent experiments that was calculated relative to the level of WT of d 0, taken as 1.

Semi-quantitative RT-PCR was used to assess expression levels of nine stress-responsive genes in both WT and the transgenic lines under normal conditions and after 7-d water stress (Figure 10). In the absence of water stress, transcript levels of *NtADC1*, *NtADC2*, *NtSAMDC*, *NtLEA5*, *NtERD10C*, *NtCDPK2 *and *NtDREB *were obviously enhanced in the two transgenic lines compared with those of WT, whereas those of *NtAREB *and *NtERF *underwent minor change. Drought stress caused upregulation of all nine genes in WT and the transgenic plants. However, it is noticeable that steady state mRNA levels of the genes in the two transgenic lines were still higher than WT, particularly *NtADC2*, *NtLEA5*, *NtERD10C *and *NtDREB*. These data indicated that overexpression of *PtrABF *in tobacco led to change in the transcript levels of endogenous stress-related genes.

## Discussion

It has been well documented that upon exposure to abiotic stresses transcript levels of a multitude of genes are altered. Numerous studies have shown that ABFs/AREBs play significant roles in regulating these stress-related genes via interaction with ABRE *cis*-element in their promoters, suggesting that ABFs/AREBs are tightly involved in plant response to various adverse environment conditions. In this work, a gene encoding ABF from *P*. *trifoliata *was isolated by RT-PCR in combination with bioinformatics approach based on ESTs deposited in the public database. Sequence multiple alignment demonstrated that *PtrABF *showed high degree of sequence identity with ABFs of other plants retrieved from the database at amino acid level. A highly conserved bZIP domain consisting of a basic region responsible for DNA binding and three heptad leucine repeats related to TF dimerization [23] was observed near the C-terminus of PtrABF, indicating that *PtrABF *encoded a bZIP family protein. Apart from the bZIP domain, it also contained four highly conserved regions at the N or C-terminus (C1, C2, C3 and C4). Within these regions, several serine (S) and threonine (T) residues or consensus sequences are present, consistent with the structure of ABFs from other plants [11,12]. These conserved residues have been suggested as phosphorylation sites of different kinases, such as calmodulin-dependent protein kinase II (R/KXXS/T, position 28-31) and casein kinase II (S/TXXD/E, position 36-39), cGMP-dependent protein kinase (K/RXXXS/T, position 49-53), implying that activation of PtrABF might be regulated by protein phosphorylation, as has been reported in other AREBs/ABFs [12,15,24,25]. Presence of these common characteristics demonstrated that *PtrABF *cloned in our study shared striking sequence similarity with other ABFs and may have a biological function same as or similar to them in abiotic stress response. The phylogenetic tree revealed relationship between PtrABF and ABFs from other plants, in which PtrABF was closely related to ABFs from dicots, including the ABFs of *Arabidopsis thaliana*. Although most ABFs in the tree remain to be characterized, *in planta *functions of ABFs of *Arabidopsis *have been determined, in which ABF3 and ABF4 act as activators of ABA/stress response. In addition, overexpression of ABF2, ABF3 has been shown to affect multiple stress tolerance, implying that PtrABF might function in the same way as ABFs of *Arabidopsis*.

An important feature of plant ABFs is the induction of their transcript levels by abiotic stresses [8,11,12,16]. QRT-PCR analysis demonstrated that steady state mRNA levels of *PtrABF *were induced by ABA, dehydration and low temperature. Expression patterns of *PtrABF *were largely similar to ABF4/AREB2 that has been shown to be induced by ABA, salt, cold and drought [11]. However, it has to be mentioned that *PtrABF *was not induced by salt, different from *ABF4*. In *Arabiopsis *it has been shown that although ABFs were all induced by ABA, they are differentially regulated by various stresses and have been suggested to play various roles in stress response [11]. ABF family members might have specificity in their role under different stresses apart from existence of function redundancy, in which ABF3 and ABF4 played essential roles in germination control and ABA/stress response, whereas ABF2 was more closely implicated in seedling growth regulation and glucose response [8,13]. Induction of *PtrABF *by both dehydration and low temperature seems to indicate that this gene may participate in response to these cues.

Compared with low temperature, dehydration caused more profound induction of *PtrABF *mRNA abundance, which compelled us to do in-depth work on elucidation of the potential role of this gene for enhancing dehydration and drought tolerance. To this end, transgenic tobacco plants were produced via *Agrobacterium*-mediated transformation of *PtrABF *under the control of CaMV 35S promoter. The two selected transgenic lines exhibited better phenotypic morphology, concomitant with less water loss, lower electrolyte leakage and higher chlorophyll content than wild type under either dehydration or long-term water stress, suggesting that overexpression of *PtrABF *conspicuously conferred tolerance to these adverse conditions. Apart from the water stress used herein, the transgenic plants also exhibited enhanced tolerance to low temperature treatment in comparison with WT (data not shown). Our work agreed with earlier reports, in which overexpression of ABF family members has been shown to render tolerance to multiple stresses in the same transgenic line [13,19-21,26], implying that ABFs class transcription factors hold great potential for genetically manipulating stress tolerance.

Despite the fact transformation of *ABF *genes led to improvement of abiotic stress tolerance, the physiological mechanism underling the tolerance remained largely unknown. This stimulated us to carry out more work to find out physiological difference between the transgenic plants and WT under stress. We put special emphasis on comparing their ROS levels because it has been well accepted that in biological systems ROS accumulation is related to physiological perturbation and ROS levels can reflect the degree of damage to cellular components [27]. Histochemical staining by DAB and NBT clearly demonstrated that under dehydration and drought conditions the two transgenic lines accumulated remarkably less O_2_- and H_2_O_2 _than WT. As ROS level during stresses greatly relies on the homeostasis between generation and removal [27], accumulation of less ROS in the transgenic lines seems to indicate that scavenging systems in these plants might work more effectively compared with WT. In order to detoxify stress-induced ROS, plants evolve a complex antioxidant system, in which several enzymes play essential roles, leading to scavenging ROS and protecting the cells against oxidative stress [27,28]. Of the enzymes, SOD provides the first line of defense against ROS by catalyzing the dismutation of O_2_- to oxygen and H_2_O_2_, which was then scavenged by coordinated action of CAT and POD [29]. In our study, activities of SOD, POD and CAT in the two transgenic lines were not profoundly different from those of wild type under well-watering conditions although they were slightly higher in the transgenic lines, which sounds reasonable because under normal conditions ROS production remained at low levels and oxidative stress was not serious [27]. However, under water stress, activities of the three enzymes were significantly higher in the transgenic plants than WT, implying that the transgenic plants had more robust detoxifying system to eliminate ROS produced during stress, which is consistent with the dramatic reduction of ROS level and ROS-associated membrane damage (lower electrolyte leakage). It is noticed that activation of antioxidant enzymes was consistent with the upregulation of the three genes, suggesting that these enzymes may be regulated at transcriptional levels. Induction of genes involved in ROS scavenging has been previously reported when TF was ectopically expressed [30], suggesting that the TF might transcriptionally regulate the expression of genes related to oxidative reactions. Our work suggested that deployment of a better ROS-scavenging system might be an integral part of defense against drought in the transgenic plants expressing *PtrABF*.

To cope with unfavorable environmental constraints plants modulate the expression of a large spectrum of stress-responsive genes, constituting an important molecular basis for the response and adaptation of plants to stresses [22,31,32]. In order to understand regulatory function of *PtrABF *and to explain the enhanced drought tolerance at molecular levels, transcript levels of nine stress-responsive genes were monitored before and after drought treatment, including five genes encoding functional proteins (*NtADC1*, *NtADC2*, *NtSAMDC*, *NtERD10C *and *NtLEA5*) and four encoding regulatory proteins (*NtAREB*, *NtCDPK2*, *NtDREB *and *NtERF*), which or whose homologues in other plants have been shown to be involved in abiotic stress response. RT-PCR analysis showed that steady-state mRNA levels of these genes were higher in the transgenic plants compared with those of WT in the absence of water stress, in line with earlier reports in which overexpression of a TF resulted in extensive alteration of transcript levels of an arsenal of related genes [33,34]. Although expression levels of all of the tested genes were upregulated by drought, they were still higher in the transgenic plants than in WT, indicating that these genes were more intensely induced in the transgenic lines. *NtADC1*, *NtADC2 *and *NtSAMDC *are genes involved in biosynthesis of polyamines, which are low-molecular-weight polycations and have been shown to be important stress molecules [35,36]. Polyamines function in stress adaptation by acting as osmoticum regulator or membrane stabilizer through binding to macromolecules like proteins, nucleic acids and phospholipids of plasma membrane [37,38]. More drastic induction of these genes implied that the transgenic plants might synthesize higher levels of polyamines to prevent them from lethal injury and maintain better growth under water stress [39]. On the other hand, polyamines have been also proposed to act as free radical scavengers [40], and the larger induction of the polyamine biosynthetic genes agreed with lower ROS accumulation in the transgenic plants after drought stress. *NtLEA5 *and *NtERD10C *encode hydrophilic late embryogenesis abundant (LEA) proteins that are assumed to play critical roles in combating cellular dehydration [41]. Higher expression levels of these genes suggested that the transgenic plants might provide more chaperones for various substrates and maintain membrane integrity or efficiently bind water, which are important strategies for plants to sustain growth during drought [41,42]. Induction of these functional genes to higher levels suggested that the transgenic plants might synthesize more protective compounds (polyamines) or proteins (LEA), which, along with others that were not identified herein, provided better adaptive or defensive niches against water stress, leading to alleviation of cellular damage when they were subjected to drought. Upregulation of the above genes suggests that they might be transcriptionally regulated by *PtrABF *through binding of the *cis*-element ABRE in their promoter. Although we could not provide evidence for this speculation, ABRE has been discovered in the promoter of most genes encoding LEA proteins [41] and in *AtADC2 *and *AtSAMDC2 *of *Arabidopsis *[43]. In addition to the functional genes, it is noted that the *NtCDPK2 *and *NtDREB *were also induced to higher levels in the transgenic lines with or without drought stress, while *NtERF *and *NtAREB *were only slightly induced upon water stress. Induction of their mRNA to higher levels raised the possibility of interaction between them and *PtrABF *in order to orchestrate well-defined stress tolerance machinery that functions in protection of the plants against adverse environment. Interestingly, the expression patterns of *NtCDPK2 *and *NtDREB *were largely consistent with those of the functional genes before and after water stress. *NtCDPK2 *and *NtDREB *are important regulatory molecules involved in signal transduction or transcriptional regulation during stress conditions [4,5,44,45]. These genes may act as the intermediates between *PtrABF *and the aforementioned functional genes. In this case, *PtrABF *might function to facilitate transcriptional upregulation of these endogenous regulatory genes, which in turn activated their downstream target genes, including those mentioned above. In the future, extra work is needed to decipher the connection between these genes so as to gain more insight into the molecular mechanisms underlying *PtrABF *function in water stress tolerance.

## Conclusions

A class-A bZIP transcription factor encoding *PtrABF *has been successfully isolated from *P*. *trifoliata*, which was induced by dehydration, low temperature and ABA. *PtrABF *was confirmed as a transcription factor due to subcellular localization in the nucleus, presence of transactivation activity and binding with ABRE.

Overexpression of *PtrABF *in tobacco enhanced tolerance to dehydration shock and long term water stress (drought). The tolerance may be ascribed to more robust activation of ROS scavenging system and coordinated and timely activation of an array of stress-responsive genes at molecular level, leading to synthesis of a wide range of protective compounds and proteins. These strategies may act in cooperation to render a more protective line of defence against the water stress.

The results presented here demonstrate that overexpression of *PtrABF *significantly modified drought tolerance in the transgenic plants, indicating that *PtrABF *might be a candidate gene with potential application to enhance abiotic stress tolerance.

## Methods

### Plant materials and stress treatments

Eight-month-old plants grown in the seedling beds at National Center of Citrus Breeding, Huazhong Agricultural University, were used to isolate the gene and to examine the gene expression patterns under different treatment. Uniform and healthy shoots were excised from the plants and inserted in a flask containing distilled water, which were kept for 1 d in a growth chamber at 25°C with 16-h light/8-h dark photoperiod (45 μmol m^-2 ^s^-1^) before exposure to the following treatment. For dehydration treatment, the shoots were placed on filter papers (90 × 90 mm) and allowed to dry for 0, 0.5, 1, 3 and 6 h. Low-temperature stress was imposed by transferring the shoots to 4°C for 0, 5, 24, 48 and 72 h. For salt or ABA treatment, the shoots were transferred to beakers containing fresh distilled water added with either 200 mM NaCl or 100 μM ABA, kept in the same growth chamber for designated time (0, 5, 24, 48 and 72 h for salt, 0, 1, 2, 3, 4 and 5 d for ABA). For each treatment, a minimum of 30 shoots were used, and leaves were sampled from three randomly selected shoots at each time point and mixed as a material pool. The leaves from all of the treatments were frozen immediately in liquid nitrogen and stored at -80°C until use for RNA extraction.

### Cloning and bioinformatics analysis of *PtrABF*

In *silico *search against HarvEST http://harvest.ucr.edu, used as a primary sequence data set, was performed to get sequences that can be merged into one contig. In order to amplify a full-length cDNA encoding ABF, total RNA was extracted with TRIZOL reagent from leaves sampled from the shoots dehydrated for 6 h. After treatment with RNase-free DNase (Promega, Madison, WI, USA) to remove genomic DNA contamination, 1 μg of total RNA was reversely transcribed into cDNA with First Strand cDNA Synthesis Kit (Toyobo, Osaka, Japan) following the manufacturer's instructions, which was then used for RT-PCR to amplify the gene. Unless otherwise stated, PCR reaction in this research consisted of 100 ng cDNA, 1 × reaction buffer, 2.5 mM MgCl_2_, 0.25 mM dNTP, 1 U of *Taq *DNA polymerase (Fermentas) and 0.5 μM of each primer designed based on the assembled contig (GSP1, Table 1) in a total volume of 25 μl. The PCR programme consisted of a 5-min incubation at 94°C, followed by 35 cycles of 30 s at 94°C, 30 s at 60°C, and 90 s at 72°C, followed by a 15-min extension at 72°C. The PCR product was sub-cloned into the pMD18-T vector (TaKaRa, Dalian, China) to get pMD18-T-*PtrABF *and sequenced (UnitedGene, Shanghai, China). cDNA and protein sequences were analyzed using the BLAST algorithm, and multiple alignments of the deduced amino acid sequence were performed using the ClustalW program. A phylogenetic relationship tree was constructed by the neighbor-joining (NJ) method using MEGA (version 4.0). Theoretical isoelectric point (pI) and molecular weight were also identified by tools in an internet server, ExPASy (Expert Protein Analysis System, http://www.expasy.org/tools). Prediction of the bZIP domain was performed on SMART (Simple Modular Architecture Research Tool, http://smart.embl-heidelberg.de/), a web-based protein database.

**Table 1:** Oligonucleotide primers used in this study

Genes	Primers	Annealing temperature (°C)	Product size (bp)	Sequences (5'-3')		
					
				Forward	Reverse	Used for
*PtrABF*	GSP1	60	1511	ATTGGAGCAAGCTGTTGCCACCGC	CGCAGCTGCCTACACTCCATGG	Amplification of full-length cDNA
*Actin*	Actin	58	107	ATTGTAAGCAACTGGGATGATA	AGAGGTGCCTCAGTGAGAAG	Quantitative real-time PCR
*PtrABF*	GSP2	57	113	CCATCACCAGTTCCTTATGTGTT	GATCTTGCAGCTGACTCTCTGTT	
*PtrABF*	GSP3	58	1363	GGCTCGAGATGGGATCTCAAATGAACTTCAAG (*Xho*I site is underlined)	AAGGATCCCGTCAGTGTCCTCCTCAAGCACAG (*BamH*I site is underlined)	Subcellular localization
*GFP*	GFP-GSP1	60	732	CGGGATCCATGAGTAAAGGAGAAG (*BamH*I site is underlined)	CGGTACCTTATTTGTATAGTTCATCC (*Kpn*I site is underlined)	
*PtrABF*	GSP4	60	1366	GGCCATGGCGATGGGATCTCAAATGAACTTCAAG (*Nco*I site is underlined)	ATAGGATCCCTACCAAGGGCCCGTCAGTGTCC (*BamH*I site is underlined)	Transcriptional activation assay
*PtrABF*	GSP5	58	160	ATTGGAGCAAGCTGTTGCCACCGC	CGCAGCTGCCTACACTCCATGG	Transgene overexpression
*CaMV35S-PtrABF*	35S-PtrABF	60	2013	CGCCGTAAAGACTGGCGAACAGTTCATACAGAGT	CGCAGCTGCCTACACTCCATGG	Confirmation of transgenic plants
*NPTII*	NPTII	56	742	AGACAATCGGCTGCTCTGAT	TCATTTCGAACCCCAGAGTC	
*NtADC1*		58	306	CTTGCTGATTACCGCAATTTATC	CCTTACTGCAGGCTTTTCATCTA	Analysis of expression levels
*NtADC2*		58	350	GCCGGCCCTAGGTTGTTGTGTAGATG	AGCGAACAACAAGAGGCAGCTGAAGCC	
*NtAREB*		58	224	TCTTCACAGCAAAAGCCTCA	GTGACCCCATTATGCAATCC	
*NtCAT*		58	151	AGGTACCGCTCATTCACACC	AAGCAAGCTTTTGACCCAGA	
*NtCDPK2*		58	267	AGGTGAGCTTTTCGATAGGATTATT	ACTTCTGGTGCAACATAGTAAGGAC	
*NtDREB*		58	503	GGACCCACTTGCTGATTCTT	GCGCCTCCTCATCCATATAA	
*NtERD10C*		58	366	ACGTGGAGGCTACAGATCGTGGTTTG	TCTCCACTGGTACAGCCGTGTCCTCAC	
*NtERF*		58	220	ACACTTCATTTTCACATTCCAACTT	TCTTCTAATTCTTGACCATGGCTAC	
*NtLEA5*		58	350	TTGTTAGCAGGCGTGGGTAT	CTCTCGCTCTTGTTGGGTTC	
*NtAPX*		58	262	CAAATGTAAGAGGAAACTCAGAGGA	AGCAACAACTCCAGCTAATTGATAG	
*NtSAMDC*		58	313	ATTGGTTTTGAAGGTTTTGAGAAG	TCACGTCTTGTACTTTGAGAGACAG	
*NtSOD*		58	238	AGCTACATGACGCCATTTCC	CCCTGTAAAGCAGCACCTTC	
*Tubulin*		58	163	TCCAGGACAAGGAGGGTAT	CATCAACAACAGGCAACCTAG	

### Analysis of the gene expression by quantitative real-time PCR (QRT-PCR)

QRT-PCR was performed according to [46] in order to evaluate expression levels of *PtrABF *under different treatment. RNA isolation and cDNA synthesis of the collected samples were performed as mentioned above. Each 10 μl of PCR reaction contained 5 μl 2×SYBR Green RealMasterMix (Applied Biosystems), 50 ng cDNA, and 0.4 μM of each primer specific for *PtrABF *(GSP2, Table 1), while *Actin *gene (Table 1) was used as internal control for expression normalization. QRT-PCR was performed in an ABI 7500 Real Time System (PE Applied Biosystems, Foster City, CA, USA) with following PCR program, 50°C for 2 min, 95°C for 10 min, followed by 40 cycles of 95°C for 15 s, and 57 (GSP2)/58 (Actin)°C for 1 min. Each sample was amplified in four independent replicates.

### Subcellular localization of PtrABF

The full-length ORF of *PtrABF *was amplified by PCR from pMD18-T-*PtrABF *with primers containing either *BamH*I or *Xho*I restriction site (GSP3, Table 1), and the amplicon was ligated into pMD18-T to get pMD18-T_B/X_-*PtrABF*. The *GFP *(AAB47998) coding region was PCR amplified from the pMD18-T vector with *BamH*I-*Kpn*I linker primers (GFP-GSP1, Table 1), and cloned into the *BamH*I and *Kpn*I sites of pMD18-T_B/X_-*PtrABF*, generating a recombinant construct of *PtrABF-GFP*, which was then inserted into the *Xho*I and *Kpn*I cloning sites of a pBI121 vector (a kind gift of Dr. Junhong Zhang) to produce a fusion construct (pBI121-*PtrABF-GFP*) under the control of the constitutive 35S promoter. After verification by sequencing, the fusion construct and control vector (pBI121-*GFP*, provided by Dr. Junhong Zhang) were introduced into *Agrobacterium tumefaciens *strain EHA105 by heat shock. Transformation of onion epidermal cells was done based on a previous method [47] and the onion cells were cultured on MS medium [48] for 2 d, followed by observation of transient expression of GFP with a universal fluorescence microscope (Olympus BX61).

### Transcriptional activation and ABRE binding assay of PtrABF in yeast

The transactivation experiment was carried out according to the manual of Matchmaker™ Two-Hybrid System 3 (Clotech, USA). PCR amplification of pMD18-T-*PtrABF *was carried out with a pair of specific primers (GSP4, in which *BamH*I and *Nco*I restriction sites were introduced), and the PCR product was double digested by *Nco*I and *BamH*I. The resultant fragment containing *PtrABF *(PtrABF_N/B_) was purified and subcloned into downstream of the GAL4 DNA-binding domain of vector pGBKT7 linearized with *Nco*I and *BamH*I to get a fusion construct (pGBKT7-*PtrABF*). This construct and pGBKT7, used as a negative control, were separately transformed into *Saccharomyces cerevisiae *strain AH109 according to the manufacturer's instructions (Clontech). The transformants were streaked on the SD/-Trp or SD/-Ade/-His/-Leu/-Trp medium supplemented with different concentration of 3-AT (0, 5 and 15 mM) and incubated at 30°C for 4 d, followed by evaluation of growth status of the transformants. In addition, the cells were also cultured on SD/-Ade/-His/-Leu medium added with both 15 mM 3-AT and 20 mM X-α-Gal for examination of *β*-galactosidase activity (Clontech).

A yeast one-hybrid assay (Clontech, Palo Alto, CA) was used to investigate if PtrABF bound to ABRE. For this purpose, PtrABF_N/B _mentioned above was fused to the GAL4 activation domain in the vector pGADT7 digested with *BamH*I and *Nco*I to get pGAD-PtrABF. A 42-bp oligonucleotide sequence containing three tandem repeat copies of 5'-GGACACGTGGCG-3' (ABRE is underlined) and restriction sites of *EcoR*I and *Sac*I was synthesized and cloned into multiple cloning sites upstream from the HIS3 minimal promoter in the pHIS2 expression vector that had been digested with *EcoR*I and *Sac*I to get the reporter vector pHIS2-ABRE. Both pGADT7-PtrABF and pHIS2-ABRE were co-transformed into yeast strain Y187, while pGADT7/pHIS2-ABRE and pGADT7-PtrABF/pHIS2 were also co-transformed as negative controls. The cells were cultured on SD/-His/-Leu/-Trp plates with or without 3-AT to identify the DNA-protein interaction.

### Transformation of tobacco and regeneration of transgenic plants

pMD18-T-*PtrABF *was restricted with *Sal*I and *Kpn*I, and the resulting product was inserted into *Xho*I-*Kpn*I sites of the binary vector pBI121 to generate pBI121-*PtrABF*, which was introduced into *A*. *tumefaciens *strain EHA105 by heat shock after fidelity verification by sequencing. Seeds of *Nicotiana nudicaulis *were sterilized for 20 s in 70% (v/v) ethanol and incubated in 2.5% (v/v) H_2_O_2 _for 7 min, followed by rinse with sterile distilled water for four times before they were sown on germination medium (GM) containing MS salts, 30 g l^-1 ^sucrose and 0.75% agar (pH 5.7). Seventy-day-old seedlings were used for transformation based on a leaf disc transformation method [49]. The infected explants were co-cultivated on GM added with 2.0 mg l^-1 ^6-BA and 0.3 mg l^-1 ^NAA for 3 d at 25°C in the dark, and then transferred on selection medium (GM, 2.0 mg l^-1 ^6-BA, 0.3 mg l^-1 ^NAA, 50 mg l^-1 ^kanamycin and 400 mg l^-1 ^cefotaxime). After selection for 30 d, the kanamycin-resistant shoots were shifted to fresh selection medium for further screening, and the resistant shoots were transferred to rooting medium (GM, 0.3 mg l^-1 ^NAA, 50 mg l^-1 ^kanamycin, 400 mg l^-1 ^cefotaxime) to induce roots. Presence of the transgene in the kanamycin-resistant seedlings was confirmed by PCR as described below. Putative transgenic T0 plants were maintained in a growth chamber with a 16 h light/8 h dark photoperiod at 25°C until they flowered and set seeds to select homozygous lines [50].

### Confirmation of the transgenic plants by PCR

Genomic DNA was extracted from T0 *in vitro *seedlings and wild type using a cetyltrimethyl ammonium bromide (CTAB)-based method. PCR reaction solution was the same as that of RT-PCR except the use of DNA template and primers specific to both *NPTII *gene and *CaMV*35S and *PtrABF *(Table 1). Amplifications were performed at 94°C for 5 min, followed by 35 cycles of 94°C for 45 s, 60 (35S-PtrABF)/56 (NPTII)°C for 45 s, 72°C for 1-2 min, and a final 15-min extension at 72°C. PCR products were electrophoresesed on 1.0% (w/v) agarose gel containing 0.5 μg l^-1 ^ethidium bromide and visualized under UV transillumination.

### Dehydration shock and drought tolerance of the transgenic plants

Seeds of the transgenic lines and wild type were sterilized and sown on germination medium as mentioned above. Some of the *in vitro *seedlings were subjected to dehydration and the others were transplanted to soil pots in a growth chamber for drought treatment. For dehydration analysis, the aerial parts of 35-d-old *in vitro *seedlings of each line were put on clean filter papers and allowed to dry for up to 90 min. Fresh weight of the samples was measured every 10 min to determine the rate of water loss relative to the initial value. Electrolyte leakage and accumulation of O_2_- and H_2_O_2 _in the leaves at the last time point were examined as described below.

The seedlings used for drought treatment were kept on flat-bottom trays to grow for 4 months with regular irrigation prior to withholding water for 21 d. Electrolyte leakage and total chlorophyll content of the plants exposed to drought for 7 d were measured, while leaves sampled at the onset of and 7 d after drought were frozen immediately in liquid nitrogen and stored at -80°C until use for analysis of enzyme activity and gene expression. Accumulation of O_2_- and H_2_O_2 _in the plants subjected to 21-d water stress was examined.

### Measurement of electrolyte leakage (EL) and total chlorophyll content

EL was measured according to [51] with slight modification. The collected leaves were stripped and placed in 25 ml distilled water, shaken on a gyratory shaker (200 rpm) at room temperature for 2 h, and the initial conductivity (C1) was measured with a conductivity meter (DSS-307, Shanghai, China). The samples were then boiled for 10 min to induce maximum leakage. After cooling down at room temperature electrolyte conductivity (C2) was measured, and the relative electrolyte leakage (C%) was calculated based on 100 × C1/C2.

Total chlorophyll was extracted and assayed as described previously [52]. About 0.1 g fine powder of leaf tissue was homogenized in 1 ml of 80% acetone and kept for 15 min at room temperature in dark. The crude extraction was centrifuged for 20 min at 10000 *i *(room temperature), and the resultant supernatant was used for assessing absorbance at 663, 645 and 480 nm with a spectrophotometer (Shimadzu UV-1600, Japan), and total chlorophyll content was computed in terms of fresh weight (FW).

### In situ histochemical localization of O_2_- and H_2_O_2_

*In situ *accumulation of O_2_- and H_2_O_2 _was examined based on histochemical staining by nitroblue tetrazolium (NBT) and 3, 3'-diaminobenzidine (DAB), respectively [39]. For O_2_- detection, the leaves from both wild type and the transgenic plants were immersed in 1 mg ml^-1 ^fresh NBT solution (prepared in 10 mM phosphate buffer, pH 7.8) at ambient temperature until appearance of dark spots. For detection of H_2_O_2_, the leaves were inserted into 1 mg ml^-1 ^fresh DAB solution (pH 3.8) prepared in 10 mM phosphate buffer (pH 7.8) and incubated in light until brown spots were observed. The stained leaves were then bleached in concentrated ethanol, and kept in 70% ethanol.

### Measurement of activity of antioxidant enzymes

For extraction of peroxidase (POD, EC 1.11.1.7), catalase (CAT, EC 1.11.1.6) and superoxide dismutase (SOD, EC 1.15.1.1), about 0.5 g of leaf sample was ground in liquid nitrogen with pre-cooled pestle and mortar, and homogenized in 5 ml of extraction buffer containing 50 mM phosphate buffer (pH7.8) and 1% polyvinylpyrrolidone. The homogenate was centrifuged at 10000 *g *for 20 min at 4°C and the resulting supernatant was collected for enzyme activity analysis. Activities of SOD, expressed as unit (U) ml^-1^, were spectrophotometrically measured using SOD Detection Kit (A001, Jiancheng, Nanjing, China) according to the manufacturer's instruction. POD activity, expressed as U mg^-1 ^FW, was assayed according to [39] with slight modification. The assay mixture in a final volume of 3.0 ml, which contained 1 ml of 0.05 M phosphate buffer (pH 7.0), 1 ml of 0.3% H_2_O_2_, 0.95 ml of 0.2% guaiacol and 50 μl of enzyme extract, was incubated for 3 min at 34°C. Activity of POD was determined based on the increase in absorbance read at 470 nm, and one unit of POD activity was defined as the increase of absorbance by 0.01 per min. CAT activity was measured by the depletion of H_2_O_2 _at 240 nm [53]. The sample solution was composed of 0.1% H_2_O_2_, 100 mM phosphate buffer (pH 7.0) and 100 μl enzyme extract in a total volume of 3 ml. The CAT activity, expressed as U g^-1 ^FW, was assessed by monitoring the decrease in absorbance at 240 nm as a consequence of H_2_O_2 _consumption, and one unit of CAT activity was defined as reduction of the absorbance by 0.01 per min.

### Semi-quantitative RT-PCR for analysis of gene expression

Semi-quantitative RT-PCR was utilized to examine overexpression of the transgene (*PtrABF*) in the transgenic lines, and to monitor transcriptional levels of nine stress-responsive genes, including *NtADC1 *(AF127239.1), *NtADC2 *(7230374), *NtSAMDC *(AB304782.1), *NtLEA5 *(AF053076), *NtERD10C *(AB049337.1), *NtERF *(AY655738.1), *NtCDPK2 *(AJ344156.1), *NtDREB*(EU727157.1), *NtAREB *(BAB61098), and three genes encoding the antioxidant enzymes, *NtCAT *(U93244.1), *NTAPX *(U15933.1, as gene coding for POD was not retrieved, it was used as a substitute) and *NtSOD *(AB093097). Extraction of total RNA and cDNA synthesis of the samples collected before and after drought treatment were done as described for cloning of *PtrABF*. Gene-specific primers for *PtrABF *(GSP5, Table 1) and the stress-related genes were designed by Primer 5.0 based on the public sequences (Table 1). RT-PCR amplification of each gene was performed using 100 ng of cDNA as a template and two corresponding specific primers with the following program, an initial 5 min denaturing at 94°C, 31 cycles of 30 s at 94°C, 40 s at 58°C, 40 s at 72°C, and a 10-min extension at 72°C. As an internal control to show that equal amounts of total RNA has been loaded, the cDNAs were also amplified with a pair of tobacco *Tubulin *gene primers (Table 1) with the same procedure. Detection of amplification products followed the procedure for PCR of genomic DNA. RT-PCR was repeated three times to ensure reliability of the data. In order to compare the expression difference quantitatively, expression level of the nine stress-responsive gene was normalized to the *Tubulin *gene by densitometric quantitative analysis using a software (Quantity One 4.6.2). Expression level of WT at the onset of water stress was set to 1, and those of the other samples were quantified accordingly.

### Statistical analysis

Dehydration and drought treatment of the transgenic lines and WT were repeated at least twice with three replicates for each line with consistent results, and the results of a representative experiment were presented, shown as mean ± SE. The data were analyzed using analysis of variance (ANOVA) by SAS software (version 8.0, SAS Institute, NC, USA), and statistical difference was compared based on Fisher's LSD test.

## Authors' contributions

XSH and JHL designed experiments and XJC provides the tobacco seeds. XSH performed the experimental procedures and drafted the manuscript. JHL revised critically the manuscript and finalized it. All of the authors read and approved the final manuscript.
